# Asphalt Materials

**DOI:** 10.3390/ma19040681

**Published:** 2026-02-11

**Authors:** Marek Iwański

**Affiliations:** Department of Construction Engineering, Faculty of Civil Engineering and Architecture, Kielce University of Technology, Al. Tysiaclecia P.P. 7, 25-314 Kielce, Poland; iwanski@tu.kielce.pl

Asphalt binder plays a key role in ensuring the required quality and durability of asphalt pavements. Therefore, it should have the most favourable physical and mechanical properties. To this end, to ensure high asphalt quality, research is being conducted to obtain a binder that is not only of high quality and has high rheological parameters but is also environmentally friendly and upholds the required durability of the asphalt pavement over a long period of use. To this end, various types of natural and synthetic materials [1,2] and modifiers [3,4,5] are added to asphalt. This Special Issue (SI), titled ‘Advances in Asphalt Materials (Second Volume)’, presents the latest results of theoretical and experimental research on asphalt modification and asphalt mixture technology. It summarizes the issues presented in the eleven publications included in this Special Issue.

Due to climate change, Warm Mix Asphalt (WMA) technology, in which various types of additives are used in asphalt [3,4,6,7,8,9,10] and asphalt foamed with water [11,12,13,14,15,16] or zeolite [17], is becoming increasingly popular. The use of water-foamed asphalt allows for producing asphalt mixtures at a temperature of approximately 100 °C, which is about 50–60 °C lower than that required for traditional technology. Initially, this technology was only used in the cold recycling process for constructing modernized asphalt pavement bases [18,19,20,21,22]. Consequently, the use of WMA technology has had a significant positive impact on reducing CO_2_ emissions and other volatile compounds released during the asphalt production process.

Another important effect of using asphalt materials in road construction with WMA technology is the reduction in the impact of high temperatures on asphalt. As a result, a slowdown in the ageing process, defined as the occurrence of physical and/or chemical changes that lead to a reduction in asphalt’s performance properties, is observed [23,24], as demonstrated in a study conducted by Tauste et al. [25]. As a result of these processes, the asphalt binder becomes stiffer and more brittle, and its adhesion to the aggregate is reduced. This causes a deterioration in the quality of the asphalt mixture from which the pavement layers are made. On the other hand, using WMA technology ensures the proper functioning of asphalt materials in the pavement structure over a long period of use.

A beneficial effect is observed when modifying asphalt with various types of materials, with one of the most effective materials being SBS polymer. Extensive research in this area has been conducted by G. Arley [1] and S. Zhang [26]. It has been shown that SBS polymer has a beneficial effect in terms of slowing down asphalt ageing [27], which significantly affects the durability of asphalt pavements. Research has also been conducted on the use of other organic and synthetic additives for asphalt modification [28,29,30,31]. Somé et al. [32] conducted extensive research on the effects of various types of organic oils on the ageing characteristics of asphalt. Comprehensive research on the use of lignin as an asphalt ageing inhibitor was conducted by Eskandarsefat et al. [33]. Studies on the use of natural and waste plastics, conducted by Nizamuddin et al. [2], Ragaert et al. [34], Silva et al. [35], and other research teams [36,37,38,39,40,41], have made significant contributions to this field. It should be emphasized that using Fischer–Trosch synthetic wax significantly improves the properties of asphalt over a wide range of technological temperatures [42].

In the field of asphalt materials, the principle of sustainable development should be introduced through constructing asphalt pavements made of a mineral–asphalt mix, adding recycled asphalt pavements (RAPs) [43,44,45,46]. Initially, mineral–asphalt mixtures with added RAPs were mainly used for constructing bases with asphalt emulsion or water-foamed asphalt [47], as well as, to a limited extent, for cement concrete production [48,49]. Currently, research is underway worldwide on the possibility of using RAPs in asphalt mixtures intended for constructing the upper layers of pavement structures [43,44,50,51,52,53,54], as well as on the use of aggregate from recycled cement concrete [55,56] and industrial waste materials such as bottom ash from municipal waste incineration, recovered asphalt shingles, and red mud [57,58,59].

Currently, one of the important areas of research for asphalt material technology involves combining experience in modifying asphalt with additives that improve its quality and slow down the ageing process. At the same time, these additives lower the technological temperatures required for asphalt mixture production by using as much RAP as possible or other materials obtained during road modernization and industrial technological processes. Future research should perform a comprehensive assessment of the use of asphalt additives and their synergy for ensuring a significant reduction in the processing temperature required for asphalt. At the same time, they should meet the requirements of a circular economy and the use of energy-efficient and environmentally friendly materials with reduced CO_2_ emissions, thus fulfilling the requirements set out in the UN Sustainable Development Goals and the European Union’s Green Deal program.
